# Study protocol: Evaluation of the ‘Flavour School’ sensory food education programme: a cluster-randomised controlled trial in UK primary school children, aged 4–7 years, to determine impact on confidence and curiosity in tasting vegetables and fruit

**DOI:** 10.1186/s13063-022-06612-2

**Published:** 2022-08-24

**Authors:** Nicholas M. Wilkinson, Srimathi Kannan, Harish Ganguri, Marion M. Hetherington, Charlotte E. L. Evans

**Affiliations:** 1 School of Food Science and Nutrition, Faculty of Environment, University of Leeds, Leeds, UK; 2 Internal Medicine, Department of Metabolism, Endocrinology, and Diabetes (MEND), Michigan Medicine, University of Michigan, Ann Arbor, MI USA; 3 Department of Nutritional Sciences, College of Natural Sciences, School of Human Ecology, University of Texas, Austin, USA; 4 University of Cumberlands, Kentucky and Research and Technology Database Team Lead, ECHO/PRISM Project Sub-Contracts from Icahn School of Medicine, New York, USA; 5 School of Psychology, Faculty of Medicine and Health, University of Leeds, Leeds, UK

**Keywords:** Diet, Health, Children, Sensory food education, Fruit and vegetables, Primary school, Intervention, Cluster-randomised controlled trial

## Abstract

**Background:**

Many children would benefit from a diet richer in vegetables and fruit. ‘Flavour School’ is a programme of ‘sensory food education’, which aims to increase children’s confidence and curiosity in exploring foods and flavours, especially vegetables and fruit. This study will conduct a cluster-randomised controlled trial to assess the outcomes of the Flavour School programme in primary school children aged 4–7 years.

**Methods:**

Four hundred plus children from 4+ schools will either complete the Flavour School programme (experimental group) or have no intervention with normal school teaching (control group), cluster-randomised within-schools, by school class. Baseline data collection will consist of video recorded behavioural observation during a tasting activity, and post-intervention data collection will repeat this activity after the experimental group have completed the intervention. Process measures will be assessed using a teacher engagement feedback questionnaire.

**Discussion:**

This study will provide causal data on the efficacy of a sensory food education intervention for increasing children’s confidence and curiosity in exploring foods and flavours, especially vegetables and fruit. This new knowledge will help educators and policy makers to make evidence based decisions on uptake of sensory food education.

**Trial registration:**

ISRCTN: 40249947 Date assigned 17 March 2020 Last edited 22 September 2021 Version 1.2

Trial Acronym OASES (Outcomes Assessment of Sensory Education in Schools)

**Supplementary Information:**

The online version contains supplementary material available at 10.1186/s13063-022-06612-2.

## Administrative information

Note: the numbers in curly brackets in this protocol refer to SPIRIT checklist item numbers. The order of the items has been modified to group similar items (see http://www.equator-network.org/reporting-guidelines/spirit-2013-statement-defining-standard-protocol-items-for-clinical-trials/).

**Table:** 

Title {1}	Evaluation of the ‘Flavour School’ sensory food education programme: a cluster-randomised controlled trial in UK primary school children, aged 4-7 years, to determine impact on confidence and curiosity in tasting vegetables and fruit
Trial registration {2a and 2b}.	ISRCTN: 40249947
Protocol version {3}	Version 1.0 01/09/2021
Funding {4}	The OASES project is funded by the European Union Horizon 2020 research and innovation programme under the Marie Sklodowska Curie Individual Fellow grant agreement No 799965. This project has received additional funding from Flavour School, a project partner and registered charity in England and Wales, No. 1178048. This funding was enabled by a donation to Flavour School, from Ella’s Kitchen (UK company no. 5183743). This funding will cover costs to participating schools of buying fresh produce for the food education intervention. Material support is also provided by Flavour School, comprising teaching resources and teacher training for the intervention.
Author details {5a}	Nicholas M. Wilkinson: School of Food Science and Nutrition, Faculty of Environment, University of Leeds Srimathi Kannan: Michigan Medicine, University of Michigan, Internal Medicine, Department of Metabolism, Endocrinology, and Diabetes (MEND). Ann Arbor, Michigan. University of Texas, Austin. Department of Nutritional Sciences, College of Natural Sciences, School of Human Ecology Harish Ganguri: University of Cumberlands, Kentucky and Research and Technology Database Team Lead, ECHO/PRISM Project Sub-Contracts from Icahn School of Medicine, New York Marion M. Hetherington: School of Psychology, Faculty of Medicine and Health, University of Leeds http://orcid.org/0000-0001-8677-5234 Charlotte E.L. Evans: School of Food Science and Nutrition, Faculty of Environment, University of Leeds https://orcid.org/0000-0002-4065-4397
Name and contact information for the trial sponsor {5b}	Trial Sponsor: University of Leeds Sponsor Reference: XXX Contact name: Dr Charlotte Evans Address: School of Food Science and Nutrition, University of Leeds, Leeds LS2 9JT Email: C.E.L.Evans@leeds.ac.uk
Role of sponsor {5c}	The OASES project is hosted by the School of Food Science and Nutrition at the University of Leeds.

## Introduction

### Background and rationale {6a}

The World Health Organization (WHO) rates diet related disease as a principle health burden in Europe [1]. Obesity and its co-morbidities, type 2 diabetes, and common cancers are amongst the greatest concerns [2, 3]. Eating disorders are also on the rise [4]. A healthy diet is associated with good physical and mental health [5]. Food insecurity and dietary inequalities are also important issues in the UK and are strongly linked to poverty and wider inequalities [6]. Diet in childhood tracks to diet in adulthood, emphasising the importance of early intervention [7–9].

One key area of concern highlighted by the WHO is low consumption of vegetables and fruit, especially vegetables. Most people do not eat the recommended minimum of 400g per day [10, 11]. Early life intake of a sufficient variety and quantity of vegetables and fruit (henceforth ‘FV’) provides access to a wide range of health-protective micronutrients and phytonutrients [12]. A FV rich diet containing at least five 80 g portions per day (for adults) is recommended by the UK National Health Service.^1^ Strong epidemiological evidence suggests that a diet rich in FV decreases risks for many health conditions including type 2 diabetes, cardiovascular disease, hypertension, stroke and some cancers [13, 14].

In the UK, children consume on average about 2.5 portions per day (half the recommended 5 portions), and many children obtain a large proportion of their energy intake from energy dense snack foods [15]. Increasing plant-based foods as a proportion of dietary intake is also widely accepted as one crucial aspect of a shift to ecologically sustainable food systems [16].

### Increasing children’s fruit and vegetable consumption

Changing children’s eating habits is challenging. Broadly speaking, trials of interventions intended to increase FV consumption have mostly shown statistically significant, but small, changes in consumption, and there is an increasing consensus that multiple systemic issues will need to be addressed concurrently to make a large impact on children’s FV consumption and wider dietary health, especially amongst the children most in need [17–20]. Preference consistently ranks amongst the top perceived barriers to FV consumption, especially for vegetables [11, 21, 22]. Repeated taste exposure is the best evidenced method for increasing liking in children [19, 23]. Non-food rewards can help, primarily by convincing children to actually taste many times [24]. Incorporating non-food tangible rewards such as stickers, alongside peer modelling and repeated tasting, in the multicomponent school intervention ‘Food Dudes’ had encouraging outcomes in increasing tasting, liking and consumption of FV [25, 26].

The development of healthy dietary variety via repeated taste exposure may be weakened by individual child eating traits such as food ‘neophobia’ (suspicion of novel foods) and ‘fussiness’ (very selective eating), which affect many young children to varying degrees, especially between 2 and 5 years of age [27–29]. Many exposures may be needed to change preferences, and exposure effects may be specific to the target vegetable [30]. Especially with younger children, it can be difficult and frustrating to persuade a reluctant child to repeatedly taste disliked foods.

Multisensory approaches could offer a less combative route to familiarisation for pre-school age children [30]. Acceptance of (initially) unfamiliar vegetables can increase with hands-on non-tasting sensory sessions in children aged 1–3 years [31] and 3–4 years [32, 33], and picture books can boost effects of sensory activities [34]. Non-taste multisensory play activities may be especially useful for neophobic or selective eaters, in providing a less threatening route to familiarisation than repeated tasting [35].

‘Sensory food education’ is a more structured approach than sensory play, originating in the ‘*Classes du goût*’ programme devised by French sensory scientist Jacques Puisais [36]. In 8–12 year old children, small improvements in willingness-to-taste have been reported [37, 38], alongside improvements in sensory discrimination and description [39], and development of preferences for more complex flavours [40]. Across these four studies, statistical significance was driven by the younger participants, and no effect on willingness-to-taste was found in Dutch school children aged 9-11 [41], suggesting that sensory food education may be most effective for younger children. In 3–6 year old Finnish children [42], and [43] both reported small but significant improvements in willingness to taste and consume FV.

In summary, as a recent review of children's food education concludes [20], despite some encouraging results, studies have mostly been either small, or based on surveys, and/or unrandomised, and so the purported benefits of ‘*Classes du goût*’ style sensory food education in schools remain to be clearly and causally demonstrated in larger behavioural outcome observation experiments and randomised controlled trials (RCTs). The OASES study will provide the first RCT of a *Classes du goût* style sensory food education intervention in the target age group (4–7 year old children) [44]. Details the study protocols for a randomised control trial of outcomes for a multi-component intervention featuring sensory food education, in Norwegian nursery children aged 1–3 years. Quantitative results of this study are not yet published at time of submission.

### Objectives {7}

The primary objective is to assess the effectiveness of the Flavour School sensory food education programme, with respect to its stated aim of “growing children’s confidence and curiosity in exploring (healthy) foods and flavours, especially vegetables and fruit”. A secondary objective is to assess the validity of automated video coding using the Noldus FaceReader, relative to trained human video coders, in our experimental context.

### Trial design {8}

The OASES trial is designed as a cluster-randomised, controlled, superiority, parallel group two-arm cohort study, with before and after observations, and pragmatic blinding (see {17a}). Outcomes measurement will focus on video recorded behavioural observation of children engaging in a tasting activity (following [45]).

## Methods: participants, interventions and outcomes

### Study setting {9}

The intervention and data collection will take place in primary schools in Greater London and Leeds, UK. Participating schools are (in Leeds) are Summerfield Primary, Swinnow Primary, Carr Manor Primary. In London, St Mary’s Catholic Primary (Hornchurch) and Woodmansterne Primary (Streatham).

### Eligibility criteria {10}

#### School inclusion/exclusion criteria

Participation is open to primary schools in London and Leeds regions, for children in reception, year 1 and year 2 (age range 4–7 years). Schools decide which of these year groups will participate in the study. Special needs schools, independent schools and very small schools (less than one class group per year group) will not be included.

#### Individual inclusion criteria

All children in experimental group classes will receive the intervention, delivered as part of regular school teaching. Children in the control group will receive normal teaching with no intervention. No particular level of spoken English is required. However, only children with opt-in caregiver consent will contribute data to the study.

### Who will take informed consent? {26a}

There will be two routes for caregiver consent for participating children. An online route will direct caregivers to a dedicated page at onlinesurveys.ac.uk, where they will receive information about the study, and can give consent for their child’s participation. However, pilots showed that many caregivers do not engage in the online route, so there will also be an in-person, on-paper consent route run by teachers in participating classes.

### Additional consent provisions for collection and use of participant data and biological specimens {26b}

Caregivers can opt-in to allow the use of their child’s video data for communications about the intervention and the study. Their child can participate regardless of this consent being giving. This trial does not involve collecting biological specimens.

### Interventions

#### Explanation for the choice of comparators {6b}

The comparator condition is normal teaching with no intervention. Though there might be advantages to an active intervention comparator, the burden on schools would be increased, making recruitment difficult. The real-world comparator is likely to be schools deciding whether to alter their normal teaching to include the intervention, so this comparator should provide a good reflection of real-world decision making context.

#### Intervention description {11a}

Flavour School is a programme of sensory food education, aimed at primary school children aged 4–7 years. Children participate in sensory activities with food (mostly FV), to learn about how their various senses contribute to their eating experiences, whilst familiarising and ‘making friends’ with healthy foods. Children also learn conversation skills and vocabulary to describe and share their sensory experiences. The programme is delivered once-weekly over one school term. Prior to the intervention, participating teachers attend a teacher training session and are provided with teaching materials. Flavour School aims to ‘grow children's curiosity and confidence in exploring foods and flavours, to support the development of healthy, happy relationships with food’. The Flavour School programme is produced and supported by the charity Flavour School (#1178084).

#### Criteria for discontinuing or modifying allocated interventions {11b}

There will be no special criteria for discontinuing or modifying allocated interventions.

#### Strategies to improve adherence to interventions {11c}

Researchers will keep in contact with participating schools and teachers throughout the trial, to ensure intervention delivery is progressing as planned, and offer support where needed.

#### Relevant concomitant care permitted or prohibited during the trial {11d}

There are no restrictions regarding concomitant care during the trial. However, schools will be instructed not to deliver any other sensory food education programmes to participating classes during the trial.

#### Provisions for post-trial care {30}

There is no anticipated risk of harm and compensation for trial participation.

### Outcomes {12}

The observational scenario is a tasting activity done in small groups (see the “Plans for assessment and collection of outcomes {18a}” section). The test activity will conducted < 6 weeks before (baseline) and < 6 weeks after intervention delivery is completed. Analysis metric for all measures is follow-up scores, adjusted for baseline, for all children.

### Primary measures

‘Curiosity and confidence’ are operationalised in terms of the following observable proxy behaviours.

1. Willingness to taste novel and familiar vegetables, legumes and fruit (supervised self-report)
2. Non-verbal ‘Enjoyment and Engagement’ (from observation of children’s facial expressions)
3. Verbal engagement (child’s speaking time and sensory vocabulary during the activity)
4. Normalised linear combination (mean) of 1, 2 and 3 as an overall measure of ‘curiosity and confidence’ in tasting

### Validation of Noldus FaceReader

1. In our experimental context, is the Noldus FaceReader software sufficiently accurate to partially replace human observers?

### Exploratory analysis

1. Liking for the offered range of foods (self-report)

### Primary measures

Children are not experimentally observed during the delivery of the intervention. All evaluation data are collected during the baseline and follow-up evaluation sessions, administered and supervised by the experimenter, before and after the intervention. See Additional file 1: Appendix 2 for more details of the coding regime for annotation of the video data.

### Willingness-to-taste (WTT)

Children are offered nine plant foods (see Additional file 1: Appendix 1 for list of foods), in a compartmentalised tray. ‘Self-reported WTT’ is simply the proportion of these that a child tastes, as indicated by the faces they draw on their My Tasting Card (see Fig. 2).

Children are instructed that nibbling and licking are included as tasting. A child who eats all of every sample therefore has the same self-reported WTT score as a child who takes tiny nibbles of all samples, though their behaviours are notably different. To finesse WTT scores such that these differences are noted, the human observers coding the video footage will also produce a ‘Gusto’ rating to summarise how enthusiastically each child tastes/eats the food samples, rating from 1 (very unenthusiastic) to 5 (very enthusiastic). These Gusto ratings will be used to weight the self-reported WTT scores (i.e. WTT = self-report WTT * Gusto). We will report both self-report WTT and WTT.

### Enjoyment and engagement—facial expression analysis

Individual video footage of a given child participating in the Flavour Explorers activity will be annotated continuously with an assessment of current facial expression. Coders assign an *affect valence* measure to the current facial expression, from − 2 (very negative) to + 2 (very positive), where 0 is neutral, updated upon changes of valence, such that a given valence measure is a time series covering the duration of that child’s Flavour Explorers session-time. See Additional file 1: Appendix 2 for more details on the coding regime. This valence time series is used to derive two measurements of behaviour which we term *Enjoyment* and *Engagement*, as defined below.

- *Enjoyment*: a quantitative measure of child's facial expression valence on average over one ‘Flavour Explorers’ data collection session. Calculated as mean valence over all timesteps.
- *Engagement*: a quantitative measure of amount of *change* in facial expression valence. To calculate this measure, we take the first derivative of valence and calculate its mean absolute value over the number of timesteps in the video recording.

### Why use these two measures?

Engaging in the tasting activity can produce strong negative-valence facial expressions, for example nervousness/fear on approaching an unfamiliar food, or when tasting sour or disliked foods. A child who mostly maintains a neutral face throughout could have a similar Enjoyment (mean valence) score as a child who swings between episodes of strongly positive and negative expressions, such that positive and negative events cancel out. However, their facial behaviour is quite different. The difference is captured by the engagement measure (which would be low for the former child and high for the latter).

*Overall measure for non-verbal enjoyment/engagement (facial expression)*: Enjoyment and engagement will be added to give a single primary measure for non-verbal enjoyment/engagement.

### Verbal engagement

Each time step of the video footage is annotated using one of 2 categories/levels of verbal behaviour: 0 = not talking; 1 = talking. These scores will then be summed across time steps, and divided by number of timesteps, to give one number describing the proportion of speaking time across the session. See Additional file 1: Appendix 2 for more details of the video coding scheme.

### Sensory vocabulary

Coders will note (with a key press) each relevant sensory word uttered by the child being observed on video. The sensory vocabulary measure is then total number of relevant sensory words across one ‘Flavour Explorers’ session, normalised by session length.

### Exploratory analysis

#### Liking for the food samples (this is an exploratory analysis)

During the Flavour Explorers tasting activity, the children draw ‘emoji style’ faces to indicate their liking for the food samples they taste (see the “Plans for assessment and collection of outcomes {18a}” section for more details). The primary purposes of this are (a) to serve as supervised self-report of willingness-to-taste and (b) to give the data collection scenario the feel of an ‘activity’, rather than the feel of a ‘test’.

The children have quite a lot of freedom in the faces they draw, and some children (especially the younger ones) are still mastering use of a pen. In pilots, the faces children drew were not always decipherable. As a result, the liking measure is likely to be rather noisy, with limited coverage, especially for children in reception class (i.e. the youngest children). There is no particular reason to anticipate significant pre-post changes in liking. Nonetheless, given we will have some data on children’s liking for the samples, it makes sense to explore this data, noisy as it may be. For these reasons, we will examine Liking as a secondary measure, and an exploratory analysis.

Liking will be calculated as the sum of all the faces drawn on a child’s My Tasting Card, where each face type is rated as follows:

- ‘frowny’ face = 0
- ‘flat’ face = 1
- ‘smiley’ face = 2

Liking will use the same statistical analysis used for the primary measures (see the “Statistical methods for primary and secondary outcomes {20a}” and “Methods for additional analyses (e.g. subgroup analyses) {20b}” sections). We will also calculate correlation between liking and WTT, as we are interested in the extent to which WTT depends on like/dislike for the foods offered.

#### Validation of Noldus FaceReader vs. human coders

Our question here is whether related studies in the future could safely use the Noldus FaceReader to partially replace the human coders in assessing children’s enjoyment and engagement in the Flavour Explorers tasting activity (or a similar activity). The FaceReader software has been validated against various benchmarks, e.g. [46, 47] and used to assess children’s expressions during activities (e.g. [48]), but our context offers some particular challenges. Our participants engage in a non-screen-based activity, so gaze direction is quite variable. Also, less training data is available for children, than for adults. In our context, we are only interested in how well the automated assessment of pre-post differences in ‘Enjoyment’ and ‘Engagement’ matches those of human observers, not the moment by moment analysis of the videos.

#### Validation questions

1. What is the mean difference, and the variability, between FaceReader and the human coders for follow-up measures of Enjoyment and Engagement, adjusted for baseline?
2. How significant are these differences given effect sizes?

#### Independent measures

- Control/intervention group
- Date of birth of child
- Sex of child
- Pupil Premium eligibility of child
- School
- School class

### Participant timeline {13}

**Fig. 1:**
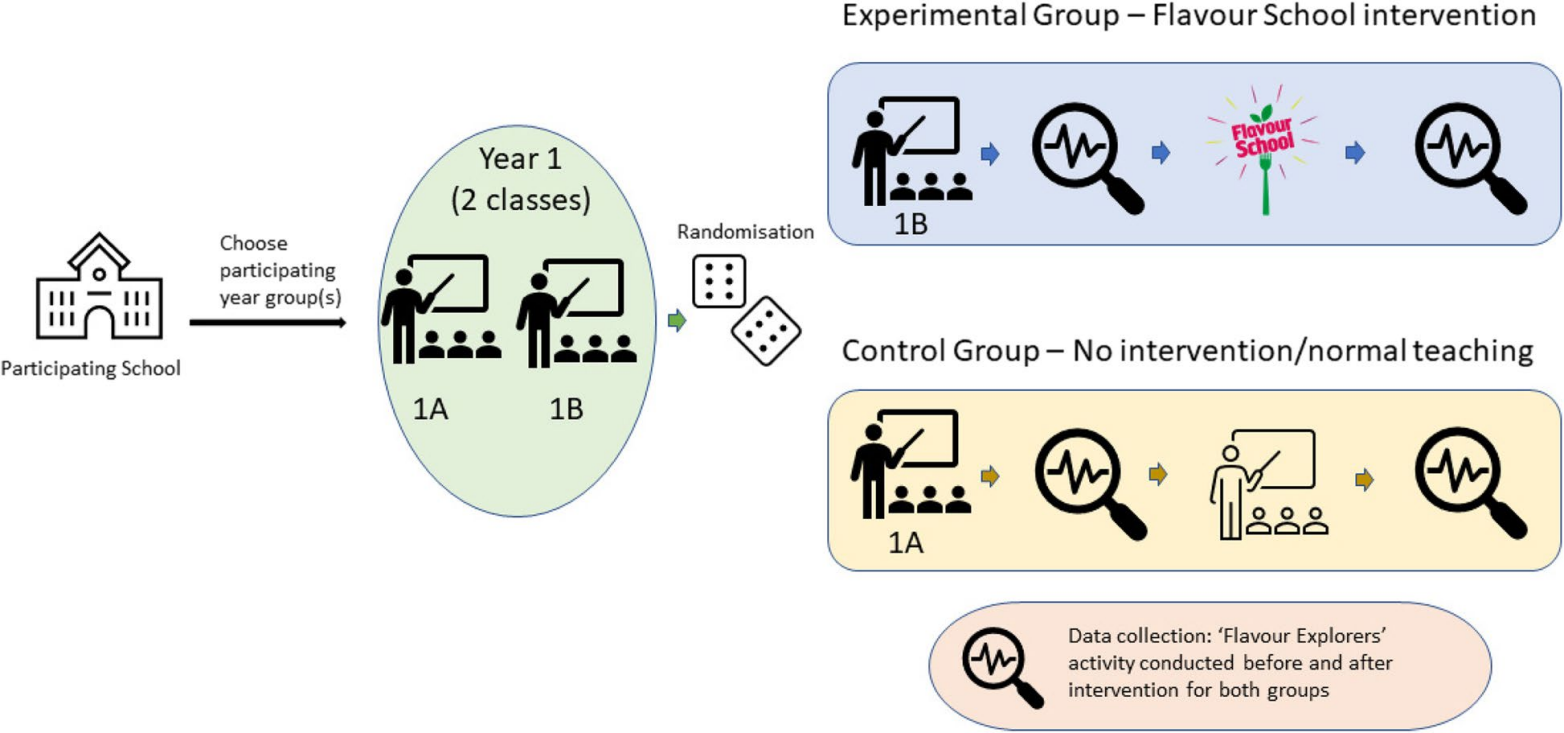
Participant timeline and cluster randomisation

The trial runs over the 2021–2022 school year. Schools nominate eligible year groups for participation. Cluster randomisation will be at the level of paired teaching class groups, within-school. For example, a school chooses year 1 to participate. Year 1 contains two classes, 1A and 1B. At random, class 1A is assigned to the control group and 1B to the experimental group. See Fig. 1. Schools can choose the timing of intervention delivery separately for each pair of classes. Therefore, different class pairs can have different intervention timings.

Teachers will be instructed to ensure Flavour School delivery respects class delineations, to minimise contagion. The control group receives no intervention (i.e. the standard curriculum). Pre- and post- intervention data collection is conducted for both the experimental and control groups. The control group can receive the intervention after post-intervention data collection is complete, at the school’s discretion.

### Sample size {14}

There is no perfectly comparable study upon which to base our sample size estimates, as our behavioural measures and study design are novel to the domain. Using a similar testing method in a younger age group (1–3 year olds), Dazeley and Houston-Price [31] reported that out of 4 foods (2 fruits, 2 vegetables) offered to nursery school children, the mean number of foods tried was 1.44 with a standard deviation of 1.38. To measure mean increases of 0.5 (the equivalent of 50% of children eating one more food), using the same standard deviation found by Dazeley and Houston Price, a sample size of 160 children is needed in each arm (control and intervention) to provide 90% power. We will aim to recruit 400+ children, but recruitment may be challenging due to ongoing pandemic COVID-19 conditions.

### Recruitment {15}

Schools will be recruited by open invitation via social media, word-of-mouth, and through local authority school-facing teams and programmes. Schools will benefit from free teacher training and reimbursement of the financial cost of the produce used for the intervention. We appreciate that this process will likely over-sample schools with an interest in food and food education. By definition, this group is the most likely to implement food education interventions, and so study results should be broadly applicable to schools choosing to introduce sensory food education. However, the results of the study may not be so broadly applicable to schools mandated to introduce sensory food education.

### Assignment of interventions: allocation

#### Sequence generation {16a}

Sequence generation will be video recorded instantaneous randomisation (die roll).

#### Concealment mechanism {16b}

None, as we will use instantaneous randomisation.

#### Implementation {16c}

The lead researcher will enrol participants. The PI will generate the random sequence and assign clusters to the experimental/control groups on the basis of randomisation.

### Assignment of interventions: blinding

#### Who will be blinded {17a}

Data analysts/video coders will be blind to condition (control/intervention) and time point (pre- or post-intervention) of a given video and participant under analysis. Due to the experiential nature of the intervention, it is not possible to blind the study participants (children and teachers) to their condition (control/intervention). The lead researcher, who will conduct data collection, will be pragmatically blinded to which classes/clusters are in which condition. However, this blinding is imperfect due to contact between the lead researcher and participants during data collection (e.g. a child might talk about doing Flavour School activities, revealing that their class is in the intervention group).

#### Procedure for unblinding if needed {17b}

**Fig. 2:**
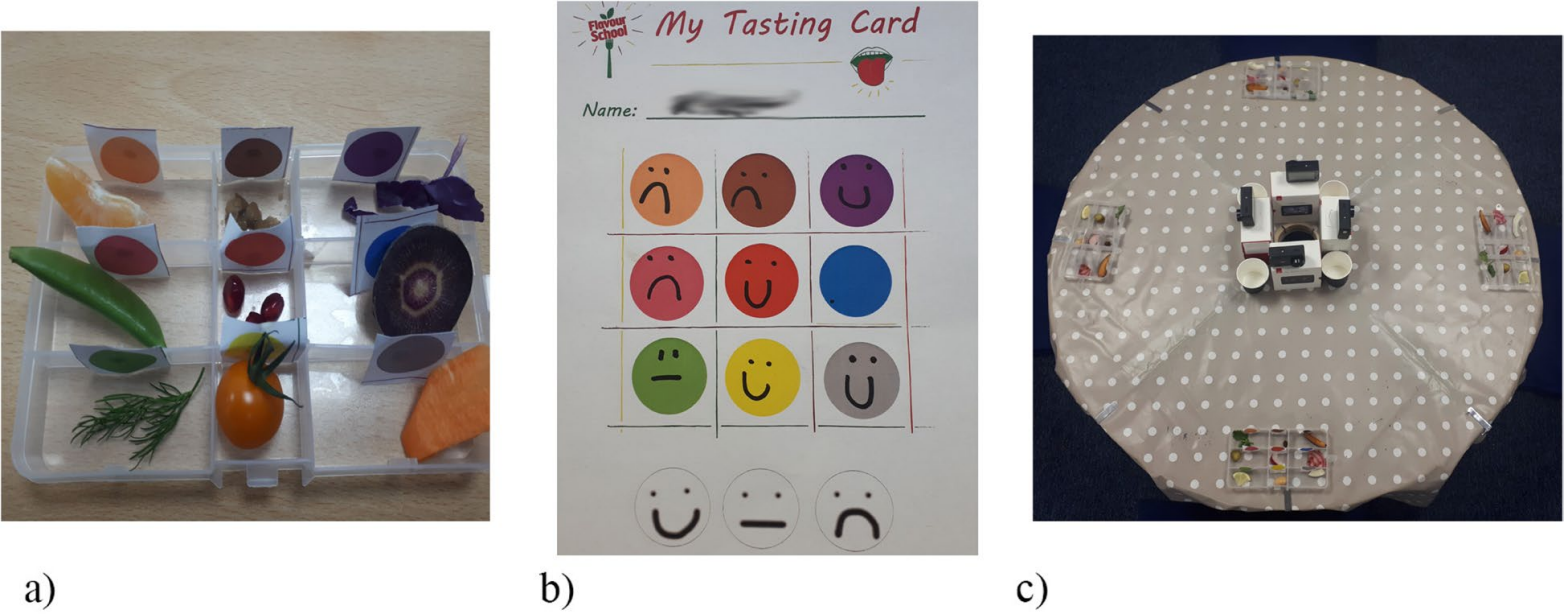
**a** Each child has a selection of FV in a compartmentalised tray. Children are free to taste (or not) any of the items, in any order. **b** Children record their tasting with an ‘emoji’ drawing to indicate like/neutral/dislike. Untasted items are left blank (e.g. blue circle). The testing table. Up to 4 chidlren participate together, each with their own tray of food samples. Located centrally on the table is a dedicated camera for each child

The lead researcher will be unblinded after the first stages of data analysis. Specifically, when all measures have been conducted, to include the WTT supervised self-report (assessed by the lead research from the children’s ’My Tasting Card’, see Fig. 2), and the independent observers have annotated the video data with their assessment of ‘Valence’ and ‘Gusto’. The project supervisor, Dr Charlotte Evans, will hold the cluster condition allocations and will inform the lead researcher at the appropriate time.

#### Data collection and management

Survey data will be collected from parents and teachers. A teacher survey (see Additional file 1: Appendix 3) will estimate teacher engagement and process adherence. The online Caregiver Consent form also contains an optional survey asking parents about their child's eating behaviours, using a subset of the Child’s Eating Behaviour Questionnaire.

#### Plans for assessment and collection of outcomes {18a}

Behavioural data collection is conducted through a tasting activity we call ‘Flavour Explorers’. The activity itself is individual, but is performed in a peer-group of three or four to enable conversation and make the scenario comfortable for the children participating. The children are supervised by the researcher(s). The children sit on cushions on the floor, around a small circular table.

Each child is provided a small tray with nine compartments, each containing one food sample. Each compartment/food sample is marked with a different colour dot sticker. Each child is also provided a ‘My Tasting Card’, with nine colour blobs matching the food samples/compartments. When a child tries a food, they use ‘emoji’ style drawings to note their liking for the food (sad face, neutral face, or happy face) on their Card. See Fig. 2. Children are given as much time as they need (within reason), and sessions typically last between 8 and 12 min.

The sample consist of seven vegetables (including a legume and a herb), and two fruits, and include both common/familiar and obscure/unfamiliar produce. Different food sample sets will be used for pre- and post-intervention testing, counterbalanced at random by school class group. See Additional file 1: Appendix 1 for more details.

**Fig. 3:**
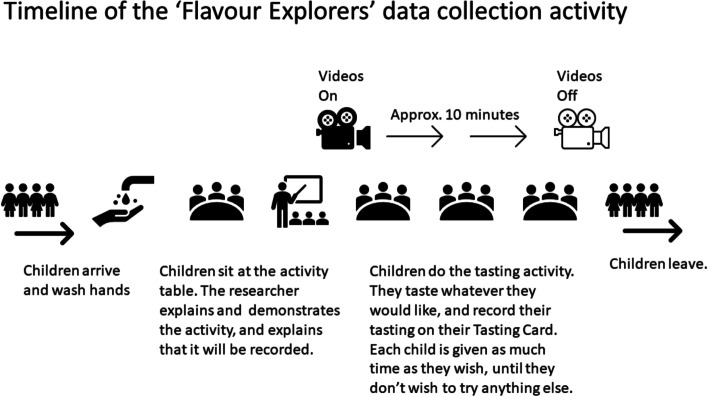
Timeline of the ‘Flavour Explorers’ data collection activity

Flavour Explorers sessions will be video recorded, with an individual video stream for each child. A dedicated Yi4k+ action camera for each child is located in the centre of the table. The My Tasting Cards provide the raw data for analysis of willingness-to-taste (WTT) and liking, whilst video/audio provides raw data for affect from facial expression, and verbal engagement (Fig. 3).

#### Plans to promote participant retention and complete follow-up {18b}

Participants who leave the school/class during the trial will not be included in the trial. The intervention is enjoyable and is integrated into school-time teaching. No follow-up measures are currently planned, due to shortening of the trial to accommodate COVID-19-related time pressures.

#### Data management {19}

The lead researcher will be responsible for all data collection.

Data entry will be conducted by the lead researcher and by the independent video observers who are annotating the video data. The latter will use the Noldus ObserverXT software to watch and annotate videos and then store their observation files on the secure School of Food Science and Nutrition server.

#### Data security

Data will be stored securely in a limited access folder in the drive for the School of Food Science and Nutrition. Data will also be backed up on an external hard drive. Some video and audio data will be shared with commercial companies who develop analysis software we plan to use. Data sharing agreements and protocols will be added to this DMP as they are completed.

Please see the OASES dynamic Data Management Plan at ISRCTN: 40249947, for up-to-date data management information.

#### Confidentiality {27}

All video data collected will be stored securely on-site at the school where it is collected, on a dedicated external hard drive, and will remain property of the school until released to the University of Leeds for use in the study. Video data will only be released to the University of Leeds after opt-in caregiver consent is obtained for a given child. The child’s video data will then be copied to another external hard drive, which will be stored securely at the University of Leeds, School of Food Science and Nutrition. Anonymous participant ID numbers will replace names in labelling participant profiles/data. All data will be stored securely, under physical locking or password protection, on secure University of Leeds servers.

#### Plans for collection, laboratory evaluation and storage of biological specimens for genetic or molecular analysis in this trial/future use {33}

**Fig. 4:**
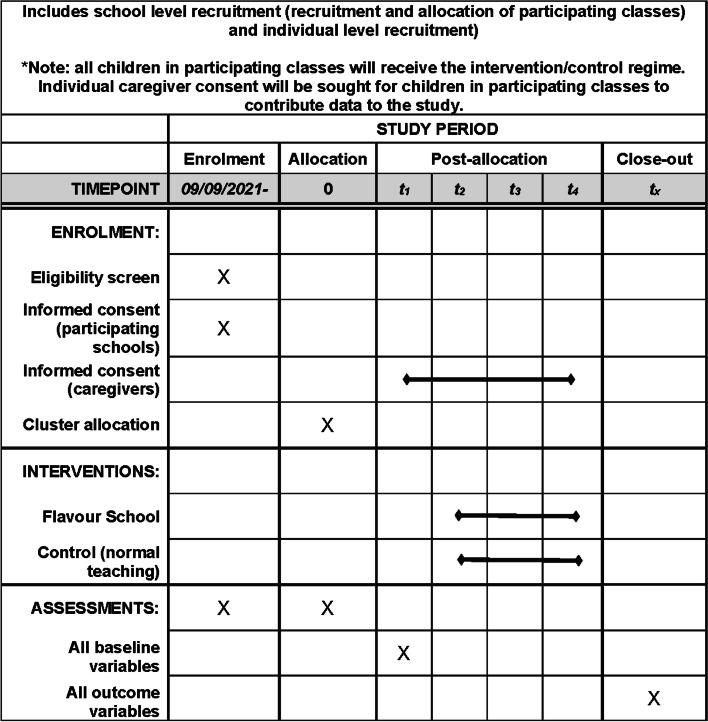
SPRIIT schedule of enrolment, interventions, and assessments

See the “Additional consent provisions for collection and use of participant data and biological specimens {26b}” section; there will be no biological specimens collected (Fig. 4).

### Statistical methods

#### Statistical methods for primary and secondary outcomes {20a}

For each participant, pre/post-intervention difference will be calculated for each primary measure, plus the normalised sum of primary measures (to represent overall ‘curiosity and confidence’). Each set of data will be analysed using regression models with pre-post difference as the outcome. Secondary measures will be examined using the same methods as primary measures. The number of clusters is too small to analyse using a multi-level model, however school and class will be included as a covariate.

For each participant, data will be collected at baseline and follow-up. The main outcomes (dependent variables) will be individual scores in WTT, enjoyment/engagement (from facial expression), and verbal engagement at follow-up. The difference in scores between the intervention and the control group will be calculated for each of the primary measures, plus an overall score consisting of the sum of primary measures which represents overall curiosity and confidence. Each set of data will be analysed using two level regression models (where appropriate), to take into account the clustering of children within schools. The analysis will be adjusted for baseline scores.

The intervention should benefit those children most in need of support. To reflect this in our analysis, we will split the participants into three sub-groups according to their score on a given measure at baseline (low, mid, high). Each grouping will cover 1/3 of the range for that measure. It is important that any benefits be observed in the low and/or mid groups and not just be driven primarily by the high group. High scores are considered desirable in all measures.

The liking secondary measure will be examined using the same regression methods.

#### Interim analyses {21b}

None. The risks of harm from the current trial and intervention are low and no greater than those for normal school teaching activities.

#### Methods for additional analyses (e.g. subgroup analyses) {20b}

At the individual level, we will control for any effects of sex, age, and Pupil Premium^2^ eligibility. At the group level, we will control for any effects of school and school class group. Any group differences will be interpreted in the light of information about process adherence and teacher engagement, as reflected in the teacher survey (see Additional file 1: Appendix 3).

#### Methods in analysis to handle protocol non-adherence and any statistical methods to handle missing data {20c}

Analysis will follow intention-to-treat principles for all participants who complete the intervention. Adherence will be assessed via a teacher survey and supported via personal contact with participating teachers and school visits by researchers to observe delivery of the intervention and offer support/guidance where necessary. A participant’s data will be excluded if it is not possible to gather post-intervention data.

#### Plans to give access to the full protocol, participant level-data and statistical code {31c}

Open access to full protocol and code is anticipated. Children’s video data will be kept private by default. Anonymised measures and statistical data will be open access via University of Leeds Library Data Service.

### Oversight and monitoring

#### Composition of the coordinating centre and trial steering committee {5d}

The co-ordinating centre is the School of Food Science and Nutrition, at the University of Leeds. The lead researcher is conducting data collection and the day-to-day running of the trial. The PI, together with a project mentor, are overseeing and supporting the lead researcher.

#### Composition of the data monitoring committee, its role and reporting structure {21a}

This is a small trial of a food education programme. Both scale and risk are small enough that a dedicated data monitoring committee has not been judged proportionate. The lead researcher will be the primary data handler, overseen by the PI. The trial is funded by an EU H2020 grant.

#### Adverse event reporting and harms {22}

Adverse events/incidents in schools will be logged through the school’s standard reporting procedures, and the study team informed to log incidents in the study risk assessment and health and safety documentation. It is not anticipated that adverse incident(s) will result in discontinuation of the study. It is possible that the trial will be discontinued or postponed due to potential disruption to schools arising from the coronavirus pandemic.

### Frequency and plans for auditing trial conduct {23}

There are no plans for auditing of trial conduct, due to the small size and low risks of the trial.

#### Plans for communicating important protocol amendments to relevant parties (e.g. trial participants, ethical committees) {25}

Changes to the protocol will be submitted to the supervising ethical committee as an amendment. Participating schools and teachers will be informed directly by email. Any changes will be submitted to Trials journal as updated versions of the protocol and to ISRCTN as updates of the OASES study account.

#### Dissemination plans {31a}

The results will be reported in scientific journals. The results will be communicated directly to participating schools. The results will be publicised via conference communications, social media and health and education networks.

## Discussion

### Novel aspects of the OASES study and their motivations

The OASES study introduces some novel measures, and methods for data collection, motivated by the desire to gain an understanding of children’s behaviour sufficient to assess any effects of the intervention on ‘curiosity and confidence’ in tasting FV. In this section, we unpack these choices.

### Data collection

Building on work with US pre-school children [32], the video recorded “Flavour Explorers” activity is designed to give us a much richer observational window on children’s tasting behaviour than simple willingness-to-taste measures. The activity typically lasts for about 10 min, during which time we continuously record video of each child individually. This allows us to observe behaviours like smiling, grimacing, laughing and talking over an extended period of tasting behaviour. Though the activity itself is individual (see the “Plans for assessment and collection of outcomes {18a}” section for more details), it is conducted in a small peer group (4 children) to enable conversation and to make the activity more comfortable and fun for the children. Doing the activity alone with a researcher/stranger might feel strange or intimidating, which in turn could impact on children’s behaviour.

### Behaviour patterns expected, based on pilots

We piloted the Flavour Explorers data collection scenario with over 200 children across the target age group and across three schools. During pilots of the evaluation methods (*with no intervention*), we observed variety in children’s engagement with the Flavour Explorers activity. Most children (~77%) tasted most or all samples (7–9). A large minority (~20%) of children were more cautious, tasting 1–6 items. A small minority of children (~2%) were very reluctant and tasted zero items.^3^

There were large differences in the gusto with which children tasted. Some gingerly licked or nibbled the samples, leaving most of each sample, whilst others consumed everything available. Some children were business-like, working their way through the samples with neither great reluctance nor great enthusiasm, perhaps a bit like completing any given piece of work at school. Others were quite animated, with multiple episodes of happy, fearful, disgust and surprise expressions. Disliking some samples often did not appear much of a barrier to tasting other samples. Children often found fun in disliking foods. Engaging in the activity can produce strong negative-valence facial expressions, for example when tasting sour or disliked foods.

We were concerned that children’s attention would be excessively drawn to the cameras filming them. However, in practice during piloting, such interest was mostly confined to the beginning and end of the sessions, with the Flavour Explorers activity mostly keeping the children engaged such that they appeared to forget about or ignore the cameras.

## Trial status

This is version 1.0 of the OASES study protocol. Date of submission is 2 November 2021. Ethical approval has been given by the Engineering and Physical Sciences Ethics Committee of the University of Leeds, project reference MEEC 18-048 AMD. Recruitment of trial centres (schools) is underway and may continue until April 2022. Recruitment of participants (in practice this means obtaining caregiver consent for use of children’s data for the study) will take place between September 2021 and June 2022.

## Supplementary Information

**Additional file 1: Appendix 1.** Details of the ‘Flavour Explorers’ activity used before and after intervention for observing children’s tasting behaviour, and gathering behavioural data. **Appendix 2.** Video coding regime. **Appendix 3.** Teacher Survey. **Appendix 4.** Caregiver consent form.

## Data Availability

Full open access is foreseen for anonymised study data, with no contractual limits. Video data of children will be kept private and limited to the research team by default.

## Abbreviations

FV: Fruit and vegetables, understood broadly to include pulses, herbs, roots, leaves, fruits
OASES: Study acronym, for “Outcomes Assessment of Sensory Education in Schools”

## Footnotes

1 UK National Health Service website https://www.nhs.uk/live-well/eat-well/why-5-a-day/ Accessed 26/10/2021

2 The Pupil Premium is a grant to schools, providing extra funding for (means-tested) disadvantaged pupils.

3 A few children (~1%) do not/cannot engage in the activity as intended and may wander off or want to leave (predominantly children with diagnoses of developmental disorders).
