# Supplementary material for: Study protocol: Evaluation of the ‘Flavour School’ sensory food education programme: a cluster-randomised controlled trial in UK primary school children, aged 4–7 years, to determine impact on confidence and curiosity in tasting vegetables and fruit
Source: Trials. 2022 Aug 24;23:705. doi: 10.1186/s13063-022-06612-2 (PMC9399583; doi:10.1186/s13063-022-06612-2)

Appendix 1 – Details of the 'Flavour Explorers' activity used before and after intervention for observing children's tasting behaviour, and gathering behavioural data

Food samples

Two different sets of food samples (Set A and Set B) will be used, each comprising 9 samples: 6 vegetables, 2 fruit and 1 pulse/legume. Set A&B will be assigned randomly to the pre- or post-intervention session and counter-balanced across the cohort, at the level of school class groups.

The food sets were chosen to include a mixture of more and less commonly eaten foods, such that most children will encounter a mixture of familiar and unfamiliar foods. They are broadly matched for food type (e.g. citrus fruit, root vegetable, herb), and chosen to present sensory variety (different colours, textures, flavours etc.). A sub-analysis will be carried out to assess whether Set A or B has intrinsic preferability (e.g. Set A foods could be more appealing overall than Set B foods). Results will be adjusted to negate any such biases.

| Set A | Set B |
| --- | --- |
| fennel | red cabbage |
| sugar snap pea | yellow cherry tomato |
| lime | orange |
| guerkin (preserved) | olive (preserved) |
| radish | purple carrot |
| candy beetroot | sweet potato |
| plum | pomegranate |
| chickpea (tinned) | broad bean (tinned) |
| coriander | dill |

Practical Instructions to give to children

The children wash/sanitise their hands, and the experimenter gives each a My Tasting Card with their name on it. The experimenter demonstrates and then guides the children into the activity with the following instructions and guidelines.

1. “Please choose a spot, and sit cross-legged on the cushion.” (Sitting cross-legged is very helpful for keeping the children in a good aspect for the video recording)

2. “You are going to be Flavour Explorers. Each of you has a little tray of foods to try, and see if you like them.”

3. “You can try anything you’d like to try, and you don't have to taste anything you don't want to – it is completely up to you.”

4. “If you do try something, you must draw a face on the matching colour blob on your card. If you like it, draw a happy face. If you don't like it, draw a sad face. If it is just ok, or you don't know, draw a flat face.” [there are printed examples of the face types on the My Tasting Card]

5. “The colour stickers on your tray match the colour blobs on you Tasting Cards. Draw your face on the matching colour.” [The researcher demonstrates three times – once for smiley face, once for neutral, and once for frowny face. Ensure the children have understood, by asking them.]

[E.g.] “I tasted the food with the green sticker. I liked it, so I’ll draw a smiley face. Where should I draw my smiley?” [‘On the green blob’.]

Keep demonstrating until you’re content that the children understand how to do the activity correctly. Use this sequence to identify any children who may need extra support during the activity.

“OK, off you go. Feel free to talk to each other about the activity.”

“We will play for about 10 minutes, or until you have tried everything that you want to try.”

“I am not going to tell you what the foods are whilst we play, but you can ask me at the end if you like.”

[Towards the end of the session] “Have you tried everything that you would like to try, or would you like a bit more time?”

Appendix 2 – Video coding regime

Note: The OASES study has limited funding for human and computer analysis, which is focussed on answering our experimental questions. The current analysis is unlikely to exhaust the richness of the video data, and further analyses may be conducted. For example, more detailed analysis of individual tasting behaviour would likely be worthwhile.

Training the coders

We will recruit 2-4 independent human coders. Coders will be trained using video footage from pilots. The research team will code 10 videos to use as guidelines/demonstrations for the coders. Using the wider set of pilot videos, the coders will practice coding with feedback from one another's ratings, until inter-coder and test-retest differences plateau at a low level. Human coders will use the Noldus Observer software for video annotation.

Annotating the video footage

Facial Expression Analysis: Non-verbal behavioural engagement and enjoyment

During the Taste Explorers activity, each child is filmed by a dedicated video camera (we use the Yi4k+ action camera), positioned in the centre of the table (see Figure 1). Average length of a video/session is about 10 minutes, but children are given as much time as they need/want, within reason. Facial expressions throughout the session will be annotated by (a) a software coder (Noldus FaceReader), and (b) human observers/coders. Facial expression is used as a proxy for internal state. For the current analysis, we are interested in the overall valence of mood/feeling (positive/negative), rather than the specific type of expression (e.g. 'sad' or 'fear' are both simply negative).

The Noldus FaceReader software coder calculates a Valence measure as follows. The software rates the presence of a set of expressions (happy, sad, angry, surprise, fear, disgust, neutral) in the subject's face, with each expression getting a percentage score. Valence is calculated as the score for Happy, minus the scores for Sad, Angry, Fear and Disgust. Noldus FaceReader considers Surprise neutral for valence purposes. However, for our purposes, we will count Surprise as positive because the activity is about exploring/tasting food, so surprise is a helpful indication of children gaining information, which we consider a positive event.

Human coders will not explicitly rate the percentage of each expression at each timestep (as the software does). They will be informed of the calculation used by the software, and told to use a similar approach, defining positive valence as characterised by Happy and Surprise expressions, and negative valence as characterised by Sad, Angry, Fear and Disgust expressions. They will use their perceptual skill and experience in reading faces, to answer the question:

'*How positive/negative does the child's facial expression look?*'

+2 Very Positive

+1 Positive

0 Neutral

-1 Negative

-2 Very negative

During video coding, the human coders will annotate changes in Valence using two control keys – one key each to nudge up or down. A third key will be used to record No Data periods (e.g. the child is out of shot, or their face is too occluded to allow for analysis). Current value of Valence is conserved over time steps unless a change is made. Therefore Valence is a time series across the (~10 minute) activity, except for periods of No Data. The primary measures for facial expressions (Enjoyment, Engagement) are functions of Valence.

NOTE: 'No Data' entries will be represented as 0/Neutral for the purposes of calculating averages, but participants with >50% No Data will be excluded from the analysis.

Comparing Human vs Noldus FaceReader

The FaceReader data gives a continuous, differentiable time series (excepting periods of No Data). The quantity we are interested in with respect to validation of FaceReader for our use case is the difference between the pre- and post-intervention measurements for a given child. Therefore, comparison of FaceReader vs human analysis will be conducted at the level of pre-post intervention differences.

Verbal behavioural engagement

Flavour School includes learning sensory vocabulary and conversation skills, to support children in sharing their food experiences. We consider that verbalisation, and richness of vocabulary used during the testing activity will be useful measures of the extent to which this aspect of the programme has affected children's verbal behaviour.

Speaking and conversation

Human coders will denote verbalisation/conversation events, using the following labels:

1. Not talking
2. Talking

Video will be annotated via two control keys, one for each state.

Relevant vocabulary richness

Human coders will press a single coding key once for each relevant food or sensory word/phrase said by the child over the session. Coders will be instructed that this measure is expected to be approximate in the case where a lot of talking occurs.

Note: During methods pilots (with no intervention), most children spoke relatively little during the Taste Explorers activity. Under these conditions, it is quite easy to code for relevant words. If children talk more post-intervention, this measure may become more approximate as the coding task becomes more onerous.

'Gusto' – summary assessment of enthusiasm in tasting

The self-report WTT measure (My Tasting Card) does not distinguish between children who chomp and chew copiously on the food samples, and children who gingerly lick or nibble them – both count as tasting. However, there are important differences between these behaviours. Therefore, the human coders will be instructed to pay attention to the tasting behaviour of the child, in terms of the quantity they consume/put in their mouth and the enthusiasm (or trepidation) with which they do so. After watching each video recording (i.e. one child in one activity session of about 10 minutes), the coder will assign a number from 1 to 5 to rate the overall 'gusto' with which the child tasted the foods. This figure will be used to weight the My Tasting Card self-reported WTT.

Appendix 3 – Teacher Survey

Teachers are asked questions in rank/scale format, with the following answer options:

1. Agree strongly

2. Agree somewhat

3. Somewhat / maybe / don't know

4. Disagree somewhat

5. Disagree strongly

The questions are in three categories:

*(a) perceived value to the children*

The children were enthusiastic about Flavour School

The children talked about Flavour School a lot

The children became more interested in food and the senses

Overall, it was difficult to engage the children in the Flavour School activities

Flavour School is a good way to teach vocabulary and conversational skills

The children learned portable behaviours, skills and knowledge

*(b) perceived value and usability to the teacher*

I enjoyed teaching Flavour School

I learned a lot through teaching Flavour School

There were many barriers to putting Flavour School into practice in my class

I plan to continue using sensory food education activities/techniques in the future

Doing Flavour School was worth the efforts required

I managed to fit in all the 12 Flavour School sessions

I would recommend Flavour School to other teachers/schools

I'd be interested in becoming a Flavour School train-the-trainer

I feel confident to design my own cross-curricular sensory food activities

Flavour School links well with other food initiatives in the school

*(c) utility/design of the teaching resources and teacher training*

The teacher training sessions were really helpful

I think I could have managed fine teaching Flavour School without teacher training, just using the teaching resources provided

The Starter Programme lesson plans/manual is clear and easy to use

The Make-it-your-own guide gives me the information and ideas I need

The Starter Programme could be better designed (please comment below)

The Make-it-your-own guide could be better designed (please comment below)

Appendix 4 – Caregiver consent form

**‘OASES’ - Outcomes Assessment of Sensory Education in Schools**

Thanks for your interest in taking part in our Flavour School research!

This form invites your consent for your child's participation at school, in University of Leeds research on Flavour School sensory food education.

**Privacy Notice**

We will securely store the personal details you provide on this form, only for the purpose of conducting the research. We will not share your personal data with anyone else. You can find the University of Leeds Research Participant Privacy Statement [linked here](https://dataprotection.leeds.ac.uk/wp-content/uploads/sites/48/2019/02/Research-Privacy-Notice.pdf).

**Information for Caregivers**

I have read and understood the Information for Caregivers - Version 1.2 (a copy of the Information for caregivers is made available)

Yes/No

**Caregiver contact details**

What is your personal/given name?

What is your family name/surname?

Please enter a contact email or postal address

**About your participating child**

What is the name of your child's school?

What is the personal/given name of your participating child?

What is their family name/surname?

What year group is your child in?

Reception

Year 1

Year 2

**Checks**

I understand that participation in this study is completely voluntary and I can change my mind at any time. Required

Yes

**Consent Form**

Please indicate whether you consent to the following aspects of participation. Consent 1 is necessary to take part. If you are not happy to consent to this, you can leave this survey by closing the browswer window. Consent 2 is optional - your child can participate whatever answer you give.

**Consent 1:** I am happy for my child’s school to transfer my child's data for the study to  University of Leeds, for the purpose of research on children's eating and tasting behaviour. I have read the Information for Caregivers, and I understand how this data will be managed and used.

Yes

**Consent 2:** I am happy for video recordings featuring my child to be used for communications about Flavour School and this research, to wider audiences (e.g. journals, conferences, websites/social media). This consent is **not** necessary for participation.

Yes

No

Maybe, but ask me first with details of use

*N.B. Parents are also requested to answer the following questions about their child's eating habits, if they are happy to. This section is optional*


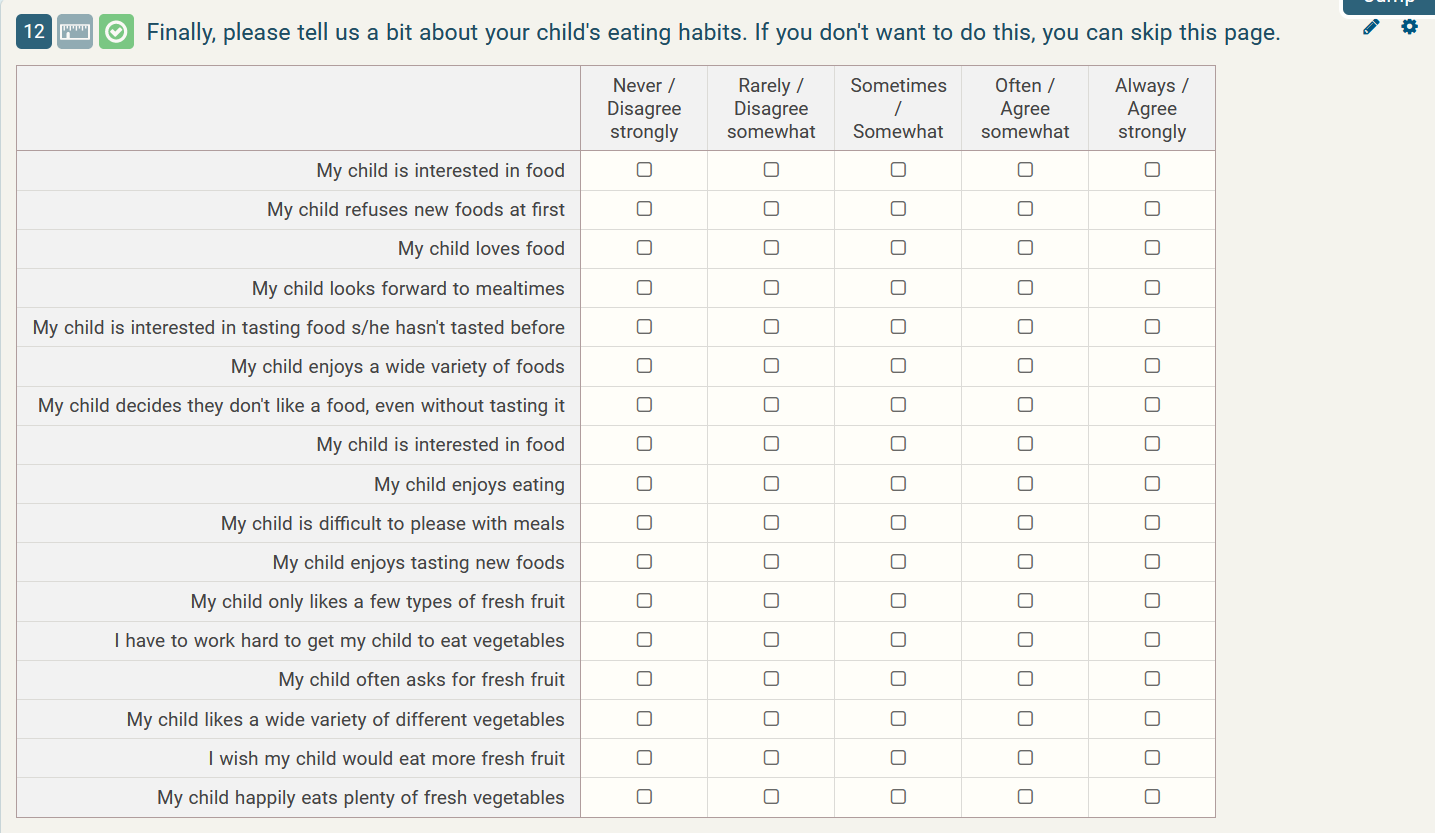

Supplement: Supplementary file 1 — Additional file 1: Appendix 1. Details of the ‘Flavour Explorers’ activity used before and after intervention for observing children’s tasting behaviour, and gathering behavioural data. Appendix 2. Video coding regime. Appendix 3. Teacher Survey. Appendix 4. Caregiver consent form. [file 13063_2022_6612_MOESM1_ESM.docx]
